# SOX10, MITF, and microRNAs: Decoding their interplay in regulating melanoma plasticity

**DOI:** 10.1002/ijc.35499

**Published:** 2025-06-03

**Authors:** Xin Lai, Chunyan Luan, Zhesi Zhang, Anja Wessely, Markus V. Heppt, Carola Berking, Julio Vera

**Affiliations:** ^1^ Biomedicine Unit, Faculty of Medicine and Health Technology Tampere University Tampere Finland; ^2^ TAYS Cancer Centre Tampere University Hospital, Wellbeing Services County of Pirkanmaa Tampere Finland; ^3^ Department of Dermatology Universitätsklinikum Erlangen, Friedrich‐Alexander Universität Erlangen‐Nürnberg Erlangen Germany; ^4^ Deutsches Zentrum Immuntherapie Erlangen Germany; ^5^ Comprehensive Cancer Center Erlangen Erlangen Germany; ^6^ DERMPATH München Munich Germany

**Keywords:** dynamic system, network biology, network motifs, phenotypic plasticity, systems biology

## Abstract

Recent studies show that the dysregulation of the transcription factor SOX10 is essential for the development and progression of melanoma. MicroRNAs (miRNAs) can regulate the expression of transcription factors at the post‐transcriptional level. The interactions between SOX10 and its targeting miRNAs form network motifs such as feedforward and feedback loops. Such motifs can result in nonlinear dynamics in gene expression levels, therefore playing a crucial role in regulating tumor proliferation and metastasis as well as the tumor's responses to therapies. Here, we reviewed and discussed the intricate interplay between SOX10 and miRNAs in melanoma biology including melanogenesis, phenotype switch, and therapy resistance. Additionally, we investigated the gene regulatory interactions in melanoma, identifying crucial network motifs that involve SOX10, MITF, and miRNAs. We also analyzed the expression levels of the components within these motifs. From a control theory perspective, we explained how these dynamics are linked to the phenotypic plasticity of melanoma cells. In summary, we underscored the importance of employing a data‐driven network biology approach to elucidate the complex regulatory mechanisms and identify driver network motifs within the melanoma network. This methodology facilitates a deeper understanding of the regulation of SOX10 and MITF by miRNAs in melanoma. The insight gained could potentially contribute to the development of miRNA‐based treatments, thereby enhancing the clinical management of this malignancy.

## Graphical abstract

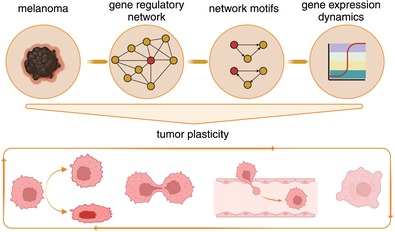

**Abbreviations**
BRAFi: BRAF inhibitors
EMT: epithelial‐mesenchymal transition
FBL: feedback loop
FFL: feedforward loop
miRNA: microRNA

## 1 INTRODUCTION

Cutaneous melanoma is an aggressive skin cancer, causing 57,000 deaths globally in 2020.^1^ Its incidence has risen, particularly among Caucasians, presenting significant healthcare challenges.^2^, ^3^ Factors such as clinical phenotype, genetic background, and UV exposure contribute to geographic variations in incidence and mortality.^4^ It is especially common in fair‐skinned individuals with light hair and eyes and is one of the most frequently diagnosed cancers in young adults.^5^, ^6^, ^7^ While the long‐term survival rate for metastatic melanoma was only 5%,^8^ advancements in BRAF/MEK inhibitors and immune checkpoint therapies have significantly improved outcomes since 2010.^9^ These treatments now enable 5‐year survival rates of 30%–50% in clinical trials for advanced cases,^9^ but mortality rates remain high and the incidence of metastatic melanoma is growing.^10^, ^11^, ^12^, ^13^, ^14^, ^15^ Therefore, advanced techniques are being used to develop complex melanoma models, improving tumor understanding and facilitating therapy development.^16^, ^17^, ^18^ Research into tumor gene regulatory networks has gained attention, emphasizing that cancer driver genes operate within interconnected signaling and transcriptional networks.^16^, ^17^ Identifying novel targets using network biology methods, which study genes within the context of their regulatory interactions, has emerged as a promising strategy for advancing cancer therapy.^19^, ^20^, ^21^, ^22^ Here, we propose and apply network biology methods to study melanoma, offering quantitative insights into the role of SOX10 and its targeting microRNAs (miRNAs) on tumor plasticity.

SOX10, a transcription factor in the HMG‐box family, plays a vital role in neural crest (NC) development and melanocyte differentiation by upregulating MITF, a key regulator of melanogenesis.^23^, ^24^, ^25^, ^26^, ^27^, ^28^ Its loss impairs NC cell migration, survival, and differentiation, highlighting its importance in early cell development.^27^, ^28^, ^29^, ^30^, ^31^ In melanoma, SOX10 is crucial for cell proliferation, migration, and cell cycle regulation, with its loss reducing proliferation but increasing invasiveness.^32^, ^33^, ^34^ It also regulates immune checkpoint proteins like HVEM and CEACAM1^35^ and modulates immunogenicity via the IRF4‐IRF1 axis.^36^ SOX10 interacts with NC factors such as MITF^37^ that is regulated by PAX3^38^, ^39^ and TFE3,^40^ forming complex networks that influence melanoma cell survival, proliferation, and metastasis.^41^, ^42^ Aberrant expression of these factors contributes to therapy resistance and tumor progression, with SOX10 facilitating the acquisition of a stemness phenotype in melanoma cells. This stemness is marked by stem cell markers, such as NES,^43^ NGFR,^44^ and PROM1.^45^ The acquisition of the stemness phenotype in melanocytes was found to be potentially dependent on the expression of SOX10, which can regulate the expression of the markers.^44^, ^45^, ^46^, ^47^ Therefore, understanding SOX10's role in melanoma could lead to novel therapies, particularly in reducing stemness properties that drive malignant transformation.^44^, ^45^, ^46^, ^47^, ^48^, ^49^, ^50^

MiRNAs are short, non‐coding RNAs that bind to the 3′‐untranslated region (3′‐UTR) of target mRNAs, repressing gene expression by affecting mRNA stability or inhibiting protein translation.^51^, ^52^, ^53^, ^54^ Some miRNAs also bind to the 5′‐UTR of mRNAs^55^, ^56^, ^57^, ^58^ or modulate gene expression through DNA methylation.^59^ MiRNAs regulate many protein‐coding genes, playing key roles in cell homeostasis and phenotypes.^60^, ^61^, ^62^ They are involved in melanoma initiation and progression through various mechanisms^63^, ^64^, ^65^, ^66^ and are linked to tumor invasion, metastasis, angiogenesis, microenvironment establishment, and immune escape.^25^, ^63^, ^64^, ^65^, ^67^, ^68^, ^69^, ^70^ MiRNAs regulate genes associated with angiogenesis, stemness, proliferation, apoptosis, and treatment resistance of melanoma,^71^, ^72^, ^73^, ^74^ making them potential biomarkers and therapeutic targets.^75^, ^76^, ^77^ miRNAs can regulate multiple malignancy‐related targets, either individually^78^ or cooperatively.^79^ Cooperative miRNAs can synergistically repress target genes, affecting tumor phenotypes like chemoresistance in melanoma cells.^80^, ^81^ Cancer genes like CDKN1A in melanoma are regulated by multiple and cooperative miRNAs.^82^ In melanoma, miRNAs collectively target genes like MITF, a transcriptional target of SOX10, to fine‐tune its expression and regulate cell proliferation and migration pathways.^83^, ^84^, ^85^, ^86^, ^87^ Here, we focus on SOX10's role in miRNA‐mediated network motifs, detailing their impact on tumor cell growth, proliferation, and migration.

## 2 THE ROLE OF MICRORNAs AND SOX10 IN MELANOMA CELL BIOLOGY

**FIGURE 1:**
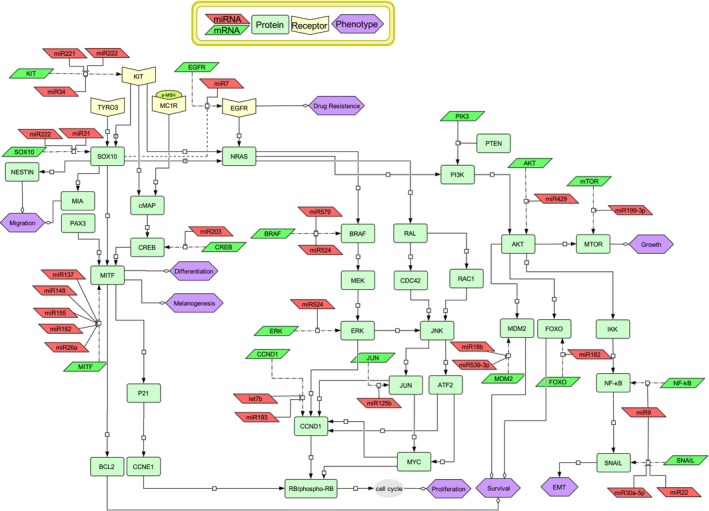
Overview of the role of SOX10 and miRNAs in melanoma. The signaling pathways and gene regulatory interactions related to melanoma were extracted from the literature and are described and discussed in the main text. The figure was created in CellDesigner, a software tool that employs a systems biology graphical notation to facilitate the exchange of graphical depictions of molecular pathways. The nodes account for molecular species and triggered phenotypes (see the figure legend). A CellDesigner file in xml format is provided in Data S3. The references used for annotating molecular interactions are in Table S1. EMT, epithelial‐mesenchymal transition.

The TF SOX10 can induce stemness characteristics in melanoma cells and sustain their proliferative and tumorigenic capabilities. However, no comprehensive review has elucidated the interactions between SOX10 and miRNAs in melanoma. Here, we provided a detailed review of studies investigating the role of SOX10, miRNAs, and their interacting molecules such as MITF that plays a multifaceted role in melanoma including regulating gene expression, influencing tumor heterogeneity, affecting immune cell attraction, and presenting potential therapeutic targets^88^, ^89^, ^90^ (Figure 1). Given their significant implications for melanoma, discussing their impact on melanoma cell phenotypes is essential. We also reviewed the current state of knowledge on the molecular interplay between SOX10, MITF, miRNAs, and other interactors in melanoma.

### 2.1 Melanogenesis

Melanoma arises from melanocytes,^91^ specialized cells housing melanosomes containing both pheomelanin and eumelanin pigments.^92^ The intricate molecular pathway governing melanogenesis begins when α‐MSH binds to MC1R, triggering elevated cAMP levels and subsequent CREB activation.^93^, ^94^, ^95^ This signaling cascade operates alongside a sophisticated transcriptional network where SOX10 and PAX3 co‐induce MITF expression, the master regulator of melanogenesis, which in turn orchestrates the expression of essential melanin synthesis genes such as TYR and DCT.^96^, ^97^ The discovery of miRNA‐mediated regulation has added another layer of complexity to our understanding of melanogenesis and melanocytic neoplasms, as these small non‐coding RNAs can modulate both MITF expression and melanogenic enzymes.^98^, ^99^ This regulatory network is exemplified by the MIR182/183/96 cluster, which targets MITF and consequently diminishes the expression of its downstream transcriptional targets (TYR, TYRP‐1, and DCT), ultimately reducing melanin production. Interestingly, when this miRNA cluster is knocked down, these genes show enhanced expression,^100^ highlighting the inhibitory role of these miRNAs in melanogenesis. A similar observation emerges with miR‐143‐5p, where its knockdown produces comparable effects.^101^ The regulatory landscape extends further with miR‐340, miR‐141‐3p, and miR‐200a‐3p, which also target MITF to influence melanogenesis.^98^, ^99^ Beyond MITF regulation, some miRNAs directly modulate melanogenic enzymes, such as miR‐203 targeting KIF5B^68^ and miR‐211 regulating TGFBR2,^102^ demonstrating the diverse mechanisms through which miRNAs control pigmentation. The interplay between miRNAs and SOX family members represents another critical axis in melanogenesis regulation. For instance, keratinocyte‐derived exosomal miR‐200 downregulates SOX1, which leads to increased nuclear β‐catenin levels and subsequent upregulation of MITF and its target melanogenesis genes TYR, TRP1, and TRP2.^103^ Conversely, miR‐155 functions as a repressor of melanogenesis‐associated genes, including SOX10, in both melanocytes and keratinocytes.^104^ Collectively, these findings underscore the pivotal and multifaceted roles of miRNAs, MITF, and SOX10 in the intricate regulation of melanocyte development and pigmentation.

### 2.2 Melanoma cell proliferation, growth, and metastasis

SOX10 emerges as a critical orchestrator of melanoma progression, with its multifaceted functions extending beyond simple growth promotion. Experimental evidence reveals that SOX10 knockdown in melanoma cells triggers profound phenotypic changes, including growth arrest, morphological alterations, and cellular senescence,^34^ highlighting its fundamental role in maintaining melanoma cell viability. The oncogenic potential of SOX10 is further demonstrated by its ability to drive the formation of giant congenital naevi and melanoma,^48^ while paradoxically, it can also inhibit melanoma cell proliferation through modulation of Notch and WNT/β‐catenin signaling pathways.^105^, ^106^ This apparent functional duality suggests context‐dependent activity that may be influenced by the tumor microenvironment or disease stage. Indeed, the expression dynamics of SOX10 and KIT during melanoma progression reveal an intriguing pattern: SOX10 expression gradually increases throughout disease progression, whereas KIT shows biphasic regulation—upregulated in primary melanoma but subsequently downregulated during metastasis.^107^ This inverse correlation during advanced disease stages points to potential mechanistic interactions that warrant further investigation. Molecularly, SOX10 upregulation closely correlates with NRAS oncogene expression, and importantly, even partial reduction of SOX10 (haploinsufficiency) significantly impedes tumor cell proliferation,^48^, ^49^ suggesting a potential therapeutic window where partial inhibition might achieve clinical benefit without complete disruption of normal melanocyte function. Mechanistically, SOX10 activates both MAPK and PI3K/AKT pathways, which are pivotal for melanoma cell survival and proliferation,^108^, ^109^ while also influencing invasive capacity, as evidenced by reduced cell invasion following SOX10 inhibition.^110^

The regulatory landscape is further enriched by miRNAs that function as tumor suppressors by modulating SOX10, MITF, and their downstream effectors in melanoma. miR‐31 exemplifies this by directly targeting SOX10, resulting in melanoma growth suppression through inhibition of the PI3K/AKT pathway^111^—a mechanism that parallels SOX10's known activation of this same pathway, creating a regulatory circuit. Similarly, curcumin‐induced upregulation of miR‐222 downregulates SOX10 levels, thereby inhibiting both tumor growth and metastatic potential through Notch signaling, which can activate MAPK and PI3K/AKT pathways while increasing N‐cadherin expression.^112^ This observation suggests potential therapeutic applications for natural compounds that modulate the miRNA‐SOX10 axis. The tumor‐suppressive miR‐107, frequently downregulated in melanoma, exerts its anti‐proliferative and anti‐invasive effects by targeting POU3F2,^113^ a transcription factor that interacts with both SOX10 and MITF in melanocytes,^114^ thus indirectly affecting the SOX10‐MITF regulatory network. Another key player, miR‐211, suppresses migration and epithelial‐mesenchymal transition by targeting KCNMA1^115^ and RAB22A,^116^ demonstrating how miRNAs can influence multiple aspects of tumor progression through diverse targets. The complexity of these regulatory networks is further illustrated by the finding that Notch signaling activation leads to MITF‐mediated de‐repression of the MIR222/221 cluster, which inhibits GRB10 and ESR1, ultimately promoting tumor invasion.^117^ Collectively, these findings underscore how multiple miRNAs, acting individually or synergistically, significantly impact melanoma metastasis and invasion through their ability to modulate SOX10, MITF, and their mutual targets via direct and indirect mechanisms.

### 2.3 Phenotype switch in melanoma

Cellular plasticity represents a hallmark feature of melanoma biology, enabling these malignant cells to dynamically transition between distinct phenotypic states—proliferative/differentiated (melanocyte‐like) and invasive/dedifferentiated (mesenchymal‐like)—as an adaptive response to environmental pressures and therapeutic challenges.^118^, ^119^, ^120^, ^121^ This remarkable adaptability is governed by distinct transcriptional programs that define each phenotypic state. The melanocyte‐like state is characterized by the expression of lineage‐specific transcription factors including MITF, SOX10, and ZEB2, alongside differentiation markers such as TYR and Melan‐A,^122^ reflecting its closer resemblance to normal melanocytes. In contrast, the mesenchymal‐like state exhibits a dramatically altered transcriptional landscape dominated by TEADs, AXL, EGFR, SOX9, ZEB1, and BRN2, coupled with heightened activity of the WNT5A and TGF‐β signaling pathways^123^—a profile more reminiscent of cells undergoing epithelial‐mesenchymal transition. At the molecular nexus of this phenotypic switching mechanism lie several master regulators, notably MITF, AXL, ROR2, and SOX10. MITF, beyond its established role in melanocyte differentiation, functions as a sophisticated metabolic sensor that integrates nutrient availability signals with appropriate metabolic responses to orchestrate specific melanoma phenotypes,^124^ demonstrating how metabolic reprogramming and phenotypic plasticity are intricately linked. The inverse relationship between MITF and AXL expression levels serves as a robust biomarker of phenotypic state, with high MITF/low AXL indicating a proliferative phenotype and low MITF/high AXL signifying an invasive phenotype.^118^ This inverse correlation suggests reciprocal regulation that may involve feedback mechanisms in regulating melanoma phenotypes. ROR2 enhances migratory capacity, EMT progression, and chemoresistance through the modulation of downstream transcription factors including AP‐1 and SNAIL,^125^ thereby promoting the invasive phenotype. SOX10, which is essential for melanocyte development, emerges as a critical mediator of phenotypic switching, as evidenced by SOX10‐deficient melanoma cells exhibiting invasive characteristics and resistance to BRAF and MEK inhibitors while paradoxically showing enhanced sensitivity to cIAP1/2 inhibitors^32^, ^122^—a finding with potential therapeutic implications for targeting resistant melanoma.

The regulatory complexity governing melanoma plasticity is further enriched by miRNAs that interact with these phenotype regulators in sophisticated ways. miR‐182 promotes an invasive phenotype through the concurrent repression of both FOXO3 and MITF,^86^ illustrating how a single miRNA can coordinately regulate multiple targets to drive phenotypic transitions. In an interesting example, MITF mediates the transcription of the MIR99A/LET7C/MIR125B cluster, wherein miR‐99a and miR‐125b promote invasiveness while let‐7c induces a proliferative state^126^—demonstrating how a single transcription factor can simultaneously activate miRNAs with opposing phenotypic effects, perhaps as a mechanism for maintaining cellular equilibrium that can be disrupted during malignant progression. Environmental stressors also influence this miRNA‐mediated plasticity, as evidenced by endoplasmic reticulum stress‐induced overexpression of miR‐410‐3p, which facilitates transition to an invasive phenotype,^127^ highlighting how cellular stress responses can trigger adaptive phenotypic shifts through miRNA modulation. Collectively, these observations underscore the pivotal roles of miRNAs and SOX10 in regulating phenotype switching, frequently through pathways involving MITF, emphasizing their intricate interplay in melanoma plasticity. Intriguingly, despite their established independent contributions to phenotypic regulation, direct interactions between miRNAs and SOX10 specifically in the context of melanoma phenotype switching remain undocumented to our knowledge—a significant knowledge gap that warrants further investigation given the potential therapeutic implications of targeting such interactions to prevent adaptive resistance.

### 2.4 Therapy resistance of melanoma

The approval of BRAF inhibitors (BRAFi) marked a pivotal advancement in melanoma therapeutics,^128^, ^129^ yet the clinical benefit of these therapies is often transient, with resistance typically emerging within 6 months due to adaptive reactivation of critical oncogenic signaling cascades, particularly the MAPK/ERK^130^ and PI3K/AKT pathways.^131^ A deeper understanding of resistance mechanisms has revealed the central role of transcriptional reprogramming, wherein silencing of SOX10 and MITF contributes significantly to therapeutic resistance. Indeed, diminished MITF expression has been documented in approximately half of recurrent cases following BRAFi treatment,^38^, ^132^, ^133^, ^134^, ^135^ suggesting that MITF downregulation represents a common adaptive response rather than a rare event. Mechanistically, SOX10 functions as a critical activator of both MAPK and PI3K pathways,^136^ and intriguingly, its reactivation can paradoxically drive resistance through promoting melanoma cell dedifferentiation,^137^ highlighting the context‐dependent role of this transcription factor. This resistance phenotype manifests clinically as decreased sensitivity to targeted therapies such as vemurafenib (a MAPK/ERK pathway inhibitor) alongside enhanced tumor cell quiescence and migratory capacity^138^—characteristics that facilitate both therapeutic evasion and metastatic progression. The regulatory network extends beyond SOX10 and MITF, as evidenced by EGFR overexpression, which occurs downstream of SOX10 downregulation and substantially contributes to BRAFi resistance.^139^ The therapeutic implications of SOX10 loss extend beyond conventional targeted therapies, as SOX10‐deficient melanoma cells exhibit resistance to oncolytic virus immunotherapy—an approach designed to reverse cancer‐associated immune suppression and stimulate antitumor immune responses.^140^, ^141^ Epigenetic alterations further influence this resistance landscape, with methylation‐mediated repression of both MITF and SOX10 promoting melanoma dedifferentiation and consequent therapy resistance.^142^ The intricate interplay between SOX10, MITF, and receptor tyrosine kinases such as AXL and ERBB3, along with their respective ligands NRG1 and GAS6, constitutes a sophisticated regulatory network governing BRAFi responsiveness,^143^ underscoring how multiple signaling nodes collaborate to determine therapeutic outcomes.

The complexity of drug resistance mechanisms in melanoma is further amplified by the involvement of miRNAs, which function as post‐transcriptional regulators of key resistance determinants.^144^, ^145^, ^146^ Intriguingly, vemurafenib treatment itself can induce upregulation of specific miRNAs, such as miR‐410‐3p, which subsequently contributes to resistance development^127^—illustrating how therapy can trigger adaptive responses that ultimately limit its own efficacy. The functional interaction between SOX10 and miR‐7 exemplifies how transcription factors and miRNAs can collaboratively modulate BRAFi response, with miR‐7 capable of reversing resistance by simultaneously targeting multiple components of resistance pathways, including EGFR, IGF‐1R, and CRAF, thereby inhibiting both MAPK and PI3K/AKT signaling.^147^ Given that SOX10 can also negatively regulate EGFR expression^139^—whose overexpression is a recognized driver of BRAFi resistance^148^—the combined repression of EGFR through concurrent overexpression of both SOX10 and miR‐7 presents a potentially synergistic approach for sensitizing resistant melanoma cells to BRAFi therapy, deserving further investigation in preclinical and clinical settings. The miRNA‐mediated regulation of therapy resistance extends beyond this axis, as evidenced by miR‐579‐3p targeting MITF^149^ and miR‐92a‐1‐5p regulating S100A9,^150^ both contributing to resistance phenotypes of melanoma through distinct mechanisms. Additionally, the downregulation of IGF1R by miR‐30a‐5p has been associated with cisplatin resistance in melanoma,^151^ highlighting how miRNA‐mediated regulation can influence response to chemotherapy. Collectively, these findings emphasize the profound impact of miRNAs on treatment resistance in melanoma, particularly through their intricate interactions with SOX10 and MITF, and suggest that therapeutic strategies targeting these regulatory networks may hold promise for overcoming resistance and improving patient outcomes.

## 3 NETWORK MOTIFS

In cancer, genes function within complex regulatory networks, forming recurrent structures called network motifs that influence cellular phenotypes.^152^, ^153^, ^154^, ^155^, ^156^, ^157^ SOX10, MITF, and other key factors in melanoma development are part of these motifs, impacting tumor progression and therapy resistance. The interaction between SOX10 and MITF links oncogenes, transcription machinery, and melanogenesis.^37^ MITF regulates melanocyte development,^158^ melanoma progression,^159^, ^160^ and immune responses,^90^ with its activity levels determining cell phenotypes. Factors like TFs (e.g., CREB, LEF1), miRNAs (e.g., miR‐101, miR‐137, miR‐218), microenvironmental stimuli,^161^ and epigenetic states regulate MITF and SOX10 expression.^88^, ^161^, ^162^

**FIGURE 2:**
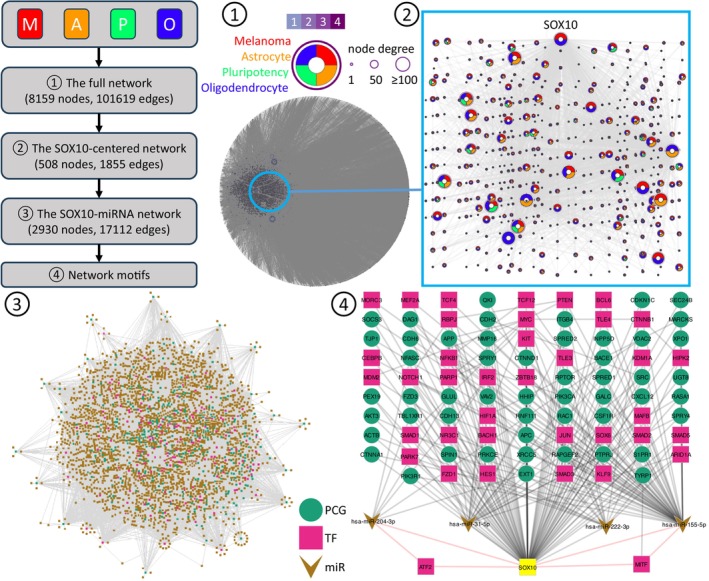
The SOX10‐mediated gene regulatory networks and network motifs. The workflow illustrates the four stages of the process used to derive network motifs. The full network was reconstructed in step 1, the SOX10‐related interactions were extracted in step 2, miRNA‐gene interactions were integrated in step 3, and three‐node network motifs were identified in step 4. In the full network and SOX10‐centered network, each node is represented by a ring diagram. This diagram indicates the origin networks of the genes associated with that node. These origin networks include melanoma, astrocyte, pluripotency, and oligodendrocyte. Additionally, the node border represents the number of networks to which the gene belongs. The greater the number of networks, the darker the border. The size of a node is proportional to the number of interacting genes, which is referred to as the node degree. In the SOX10‐miRNA network and network motif networks, the nodes are color‐coded according to their molecular species, with protein‐coding genes (PCGs) depicted in green, TFs in red, and miRNAs in brown. SOX10 is highlighted in yellow. The two network motifs (i.e., MIR155‐SOX10‐MITF and MIR204‐SOX10‐ATF2) that are discussed in the main text are also highlighted. A PDF file containing all the networks is available in Supporting Information. A Cytoscape file containing all the networks is available in Data S2.

To identify and study miRNA‐mediated motifs, we used a systems biology approach, in which we reconstructed a SOX10 network, identified miRNA‐mediated network motifs, analyzed the gene expression dynamics in those motifs, and provided them biomedical explanations related to melanoma. Specifically, we assembled a gene regulatory network considering SOX10's role in multipotency,^163^, ^164^ glial cell differentiation,^163^, ^165^ and melanoma plasticity.^32^, ^158^, ^166^, ^167^ This network integrates interactions from melanoma,^168^ pluripotency,^169^ and neural crest cells like oligodendrocytes^170^ and astrocytes (in‐house data), providing a comprehensive regulon landscape (Figure 2). We focused on SOX10's direct interactions and expanded them using experimentally validated miRNA‐gene interactions from miRTarBase.^171^ Then, we annotated the SOX10‐centered network with known human TFs.^172^ Using the network, we identified feedforward loops (FFLs) and feedback loops (FBLs) involving SOX10, MITF, and miRNAs. FFLs influence target activity via two routes, suppressing noise and inducing adaptation.^154^, ^173^ FBLs involve reciprocal regulation, leading to bistability and oscillation.^154^, ^173^ Our network analysis identified feedback and feedforward motifs involving SOX10, MITF, miRNAs, other genes that were (differentially) expressed in melanoma. We hypothesized that they may play a crucial role in melanoma malignant phenotypes and progression. The following sections elaborate on key gene expression dynamics and their potential in regulating phenotypic plasticity for the most prominent identified network motifs.

### 3.1 TFs as network hubs in melanoma: The SOX10‐mediated MITF gene expression

The SOX10‐centered network includes TFs that act as network hubs,^174^, ^175^ crucial for the network's structure and function (Figure S1 and Table S2). Hub TFs, such as SP1, HIF‐1, MYC, and KLF5, function as master regulators, controlling gene expression and driving critical cancer‐related processes like proliferation, metabolism, and immune modulation.^175^, ^176^, ^177^ These hubs interact to fine‐tune transcriptional programs, such as with FOXM1 and CENPF, which synergistically regulate target genes to promote tumor growth.^178^

**FIGURE 3:**
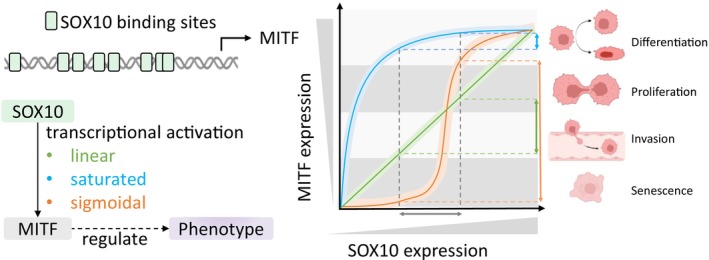
The dynamics of SOX10‐mediated MITF expression. The cartoon illustrates the binding sites of SOX10 in the MITF promoter. The diagram illustrates the interaction between SOX10 and MITF, wherein the dynamics of SOX10‐mediated MITF expression are proposed in three models: linear (green), saturated (blue), and sigmoidal (orange). The expression levels of MITF affect its downstream genes and determine tumor phenotypes (as elucidated by the rheostat model,^160^ including senescence, invasion, proliferation, and differentiation. In the dynamic plot, the solid thin lines represent the average behavior of tumor cells observed in the bulk data, while the shaded thick lines represent the heterogeneous behavior of individual cells observed in the single‐cell data. The change in SOX10 expression level (the horizontal grey arrowed line) has different effects on MITF expression levels (the vertical‐colored arrowed lines) in different models. The length of the vertical arrowed lines indicates the sensitivity of MITF to the change in SOX10 expression level, which varies depending on the model type and the actual SOX10 expression level. The tumor phenotypes are separated by gray zones, and the zone size may vary for individual cells due to their genomic profiles.

In our network, the most pertinent hubs are SOX10 and MITF, as both TFs play a crucial role in melanoma. Furthermore, SOX10 can directly bind to the MITF promoter to activate its transcription, regulating genes associated with cancer hallmarks.^179^, ^180^, ^181^ Theoretical models of SOX10‐MITF regulation propose three dynamics: linear, saturated, and sigmoidal (Figure 3). The linear model suggests a proportional relationship between SOX10 and MITF levels.^160^ However, SOX10's multiple binding sites on the MITF promoter may induce nonlinear dynamics, particularly when interacting with other TFs like PAX3.^23^ The saturated model describes a gradual diminishing response in MITF expression with increasing SOX10 levels. This is commonly observed in TF‐regulated gene expression dynamics since an excess of TFs may not have access to their binding sites in the promoter of target genes.^182^, ^183^ The sigmoidal model highlights cooperative interactions of SOX10 due to its multiple binding sites at the MITF promoter.^170^ This can lead to modest and sharp transitions in MITF expression at extreme and intermediate SOX10 levels, respectively.

MITF expression is critical for determining melanoma phenotypes and contributes to tumor cell plasticity. Dynamic phenotypic switching enables melanoma cells to alternate between migratory and invasive states depending on tumor progression^160^ and anti‐cancer treatment.^184^ Single‐cell studies reveal heterogeneity in MITF and SOX10 expression, correlating with distinct phenotypes in primary melanoma.^185^ This variability can be explained by two factors (Figure 3). (i) Differential sensitivity to SOX10: Tumor cells with varying SOX10 expression levels exhibit unique responses, leading to distinct changes in MITF expression, particularly under nonlinear regulation.^186^ Cooperative interactions between SOX10 and other TFs can further refine MITF expression at the single‐cell level through mechanisms like enhanced DNA binding or combinatorial activation. Specifically, direct TF‐TF interactions prior to DNA binding and TF‐DNA binding can increase the binding affinity of another TF.^187^, ^188^ (ii) Genomic profile variations: Individual tumor cells may follow different regulatory models (i.e., linear, sigmoidal, or saturated) based on unique genomic profiles, influencing MITF responses and defining specific tumor phenotypes. This hypothesis aligns with findings that seemingly identical cells exhibit different dynamic trajectories, resulting in different fates like survival or death.^189^ Furthermore, the range of MITF expression levels that define specific tumor phenotypes may also vary between cells. The interplay of these factors contributes to the intricate SOX10‐mediated MITF regulation underlying tumor heterogeneity.

### 3.2 Feedforward loops as fine‐tuner for regulating gene expression: The MIR155‐SOX10‐MITF circuit

**FIGURE 4:**
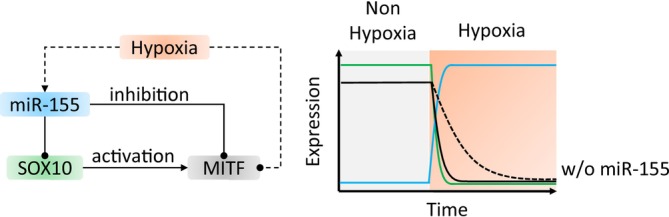
Prevention of transcript leakage regulated by the MIR155‐SOX10‐MITF FFL. The left figure shows the detailed gene regulation in the MIR155‐SOX10‐MITF coherent FFL. The oval‐head and arrow‐head solid lines represent direct inhibition and activation of genes, respectively, while the dashed lines represent indirect gene regulation in hypoxia. The right plot shows the dynamics of FLL gene expression. The occurrence of hypoxia leads to the upregulation of miR‐155, which can inhibit both SOX10 and MITF, resulting in the rapid degradation of MITF. In comparison, without miR‐155 regulation, the leaky transcripts of MITF will degrade slowly based on its half‐life.

Gene expression is controlled by a complex network of regulatory factors, including TFs that bind to DNA and miRNAs that interact with mRNA.^190^, ^191^ In this combined transcriptional and post‐transcriptional regulation, we identified network motifs like FFLs where TFs and miRNAs co‐regulate gene expression levels.^192^ miRNA‐mediated FFLs can be coherent or incoherent, based on the interactions between components.^193^, ^194^, ^195^ Coherent FFLs have consistent direct and indirect actions, while incoherent ones have opposite actions. In our network, we searched for three‐node network motifs containing SOX10 and miRNAs (Table S3) and identified a coherent FFL involving SOX10, MITF, and miR‐155 in melanoma (Figure 4). In this FFL, miR‐155 represses MITF^196^ and SOX10,^197^ while SOX10 upregulates MITF.^93^ In the following, we elaborated on the dynamic features of this FFL regulating SOX10 and MITF expression.

#### 3.2.1 Stability in gene expression

MiRNAs can precisely tune their target genes' expression levels.^82^, ^198^, ^199^ This regulation is sophisticated when a miRNA targets a TF and both share a common target gene, forming a FFL. Such FFLs fine‐tune and stabilize target gene expression.^192^ For example, the MIR17‐E2F1‐RB1 coherent FFL stabilizes RB1 expression against E2F1 fluctuations by increasing RB1 protein's response time to reach its steady state.^192^ This mechanism ensures robust cell cycle transition. Synthetic miRNA‐based FFLs can stabilize transgene expression, yielding consistent protein output despite over two‐fold variations in the underlying gene dosage or transcription rate. This stability makes such FFLs promising for gene therapy.^200^ Similarly, in the MIR155‐SOX10‐MITF FFL, miR‐155 may prolong the time required for producing the protein products of MITF or stabilize MITF expression against sudden activation or deactivation of SOX10 transcription to ensure desired melanoma cell phenotypic transitions.

#### 3.2.2 Prevention of leaky gene transcript

The other function of the FFL is to quickly degrade unwanted MITF transcripts via direct and indirect repression by miR‐155 (Figure 4).^201^ Specifically, hypoxia reduces MITF expression in melanoma cells via HIF‐1α^202^, ^203^ and upregulates the expression of miR‐155.^204^ The upregulation of miR‐155 leads to the involvement of the MIR155‐SOX10‐MITF FFL, which can prevent translation of leaky MITF transcripts (i.e., already transcribed MITF mRNA). Compared to the regulation without miR‐155, a negative regulator of melanoma cell proliferation and survival,^205^, ^206^ the leaky MITF transcripts are translated into proteins, resulting in a possible longer retention of tumor cells in aggressive phenotypes that favor tumor progression.

#### 3.2.3 Sign‐sensitive delay

**FIGURE 5:**
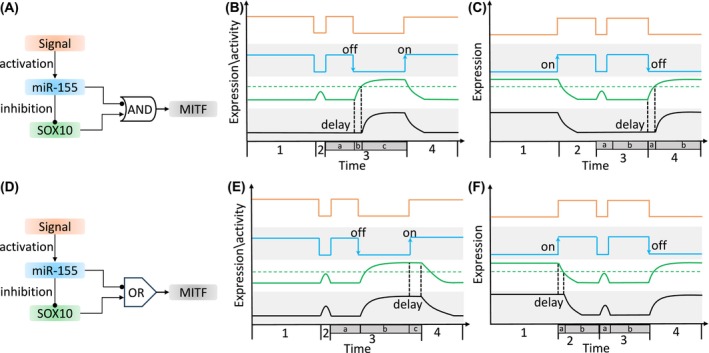
The dynamics of gene expression levels in the MIR155‐SOX10‐MITF FFL. (A) The coherent FFL with an AND gate function for MITF demonstrates a delay following signal removal (represented by the “off” state at phases 3b and 4a in (B) and (C), respectively). Furthermore, the FFL functions as a sign‐sensitive filter, responding only to persistent signals and not to transient ones (represented by phases 2 and 3a in (B) and (C), respectively). (D) In contrast, the FFL with an OR gate function for MITF exhibits a delay after signal amplification (indicated by the “on” state at phases 3a and 4 in (E) and (F), respectively) and responds to both transient (phases 2 and 3a in (E) and (F), respectively) and persistent signals. The colors of the lines correspond to the colors of the components shown in the network motif. The dashed green line indicates the threshold for SOX10 expression level to activate MITF transcription. The numbers on the x‐axis are associated with the four phases, which are delineated by segmentations indicated by alphabetic characters. The four gray zones are employed to differentiate the plot areas of the FFL components. To facilitate a more comprehensive understanding of the dynamics of the FFL, a truth table for each plot is provided in Figure S2.

In the MIR155‐SOX10‐MITF FFL, MITF is coherently regulated by miR‐155 through direct and indirect repression (Figure 5). Theoretically, the regulation of MITF expression can follow two input functions: an AND or an OR gate.^193^, ^194^, ^207^ The AND gate model requires miR‐155 to reduce MITF transcription via SOX10 and post‐transcriptionally repress MITF for effective repression. This model is based on the assumption that endogenous miRNA upregulation has mild repressive effects.^208^, ^209^ The OR gate model assumes exogenous miR‐155 upregulation can efficiently repress MITF either directly or through SOX10 downregulation.

In the AND gate model, effective MITF regulation by miR‐155 requires both direct targeting and indirect regulation via SOX10 (Figure 5A). In this model, the dynamics of MITF expression can be delayed when miR‐155 is turned off by an upstream signal (Figure 5B). Initially, high miR‐155 expression results in low SOX10 and MITF levels (phase 1). When the signal is temporarily removed, the downregulation of miR‐155 transiently upregulates SOX10 (phase 2), but MITF remains unchanged. This is because the transient upregulation of SOX10 does not cross the activation threshold required for MITF transcription. Thus, the AND gate ensures stability of MITF expression during a transient downregulation of the upstream signal. Sustained signal removal leads to miR‐155 downregulation and MITF derepression (phase 3), but MITF expression is delayed due to the AND gate function that requires SOX10 to cross the activation threshold. The length of the delay can be determined by biochemical parameters of SOX10. For instance, the higher the activation threshold for the MITF promoter by SOX10, the longer the delay. When signaling resumes (phase 4), miR‐155 upregulation silences MITF by repressing its expression at both the transcriptional (via SOX10 repression) and post‐transcriptional levels, synchronizing SOX10 and MITF dynamics. This phenomenon is a sign‐sensitive delay occurring when the signal is turned off, not on. Similar effects occur with low initial miR‐155 levels (Figure 5C) and the delay in MITF expression occurs when the signal is fully turned off (phase 4). Studies have shown that TGF‐β can modulate miR‐155 expression depending on the cell type and tissue environment.^152^, ^210^, ^211^, ^212^ TGF‐β is involved in tumor progression and EMT by regulating the MIR155‐SOX10 axis.^197^, ^213^ The AND gate FFL suggests that transient changes in TGF‐β can modulate miR‐155 and SOX10, but MITF is resistant to transient changes, requiring sustained TGF‐β signaling for expression changes in MITF that occur with a delay compared to SOX10 upregulation that causes phenotype switching of tumor cells, for example, from an invasive to a proliferative phenotype.^48^, ^214^

In the OR gate model, MITF modulation requires either direct or indirect regulation by miR‐155 (Figure 5D). This model delays MITF expression changes when the signal turns miR‐155 on (Figure 5E). High initial signal levels result in high miR‐155 and low SOX10 and MITF levels (phase 1). Temporary signal removal upregulates SOX10 and MITF (phase 2), with MITF responding immediately due to the OR gate function, assuming that removal of miR‐155 repression is sufficient to allow MITF to regain its expression. Persistent miR‐155 downregulation synchronizes MITF with SOX10 (phase 3). When the signal resumes, miR‐155 upregulation downregulates SOX10 (phase 4), and the time it takes for SOX10 to fall below the activation threshold delays MITF downregulation. Such delay effect may also occur when the signal is off, then on, and off again (Figure 5F). It has been shown that miR‐155 is upregulated in response to various stimuli, including infection,^215^ inflammatory signals,^215^, ^216^ and hypoxia.^204^ In the OR gate FFL, this leads to dynamic MITF expression via SOX10 with two scenarios. When the signal that activates miR‐155 is turned off (Figure 5E), there is an immediate upregulation of MITF that induces a more malignant phenotype. For example, upregulated MITF protects melanoma cells from apoptosis^217^ and activates genes associated with cell cycle and differentiation.^97^ When the signal is turned on (Figure 5F), a delayed MITF response maintains the malignant state of the tumor, potentially promoting tumor progression.

### 3.3 Feedback loops underlying melanoma aggressiveness: The SOX10‐MIR204‐ATF2 circuit

**FIGURE 6:**
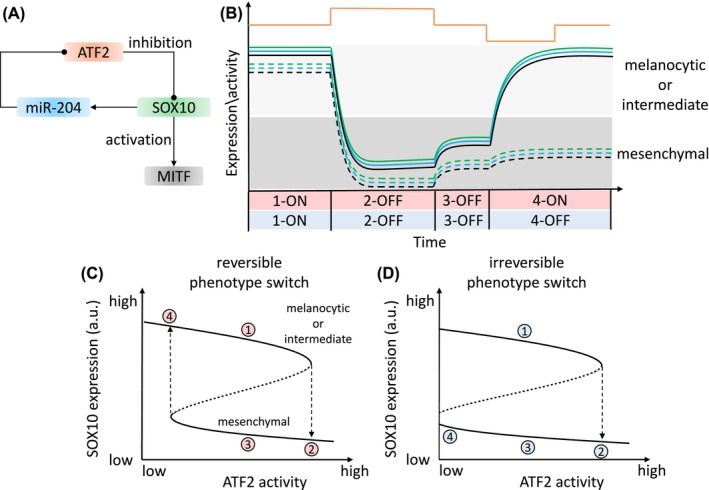
The bistability of gene expression in the MIR204‐SOX10‐ATF2 FBL. (A) The diagram shows possible molecular interactions in the SOX10‐miRNA FBL. (B) The dynamic plot shows the change in activity or gene expression levels in the FBL over time. For simplicity, we assumed that the dynamics of miR‐204 and MITF are like SOX10 because SOX10 positively regulates their transcription. ATF2 is shown at the top of the plot. The colors of the lines correspond to the color of genes shown in the network motif. The solid and dashed lines correspond to the scenarios shown in bottom left and right panels, respectively. The background colors indicate phenotypic states of melanoma cells that is determined by the expression level of MITF. The numbers on the x‐axis correspond to the four phases described in the main text and they also correspond to the numbers in the bottom plot. The terms “ON” and “OFF” indicate the upper and lower steady states of SOX10, respectively. The bifurcation plot illustrates the reversible (C) and irreversible (D) switching between melanoma cell states. The solid and dashed parts of the curves represent stable and unstable steady states of SOX10, respectively. The upper and lower steady states correspond to ON and OFF steady states in the middle plot. The numbers in red and blue circles indicate the dynamics of gene expression and follows the order as shown in the middle plot (i.e., 1 > 2 > 3 > 4). The ability of melanomas to revert to their initial cell states is dependent on the location of the left bifurcation point. If the left bifurcation point is located within the activity interval of ATF2, the tumor cells can switch back to their initial states. If the left bifurcation point is in a position that lacks biological meaning (e.g., a negative value of ATF2 activity), melanomas become trapped at the lower part of the curve and cannot return to their initial states. For a more detailed theoretical explanation about bifurcation points, please refer to Reference 238.

In addition to FFLs, FBLs are network motifs in gene regulation and signal transduction networks underlying tumor genesis and progression.^218^ FBLs involve downstream molecules promoting (positive) or inhibiting (negative) upstream processes. Negative FBLs maintain homeostasis by balancing noise and preventing overwhelming activation.^154^, ^173^ Positive FBLs amplify signals or induce switch‐like behaviors in gene activation. In our network, we identified an FBL involving miR‐204, ATF2, and SOX10 that regulates MITF expression (Table S3). In the FBL, miR‐204 inhibits ATF2 post‐transcriptionally,^219^ ATF2 inhibits SOX10,^220^ and SOX10 upregulates miR‐204 transcription.^219^ In melanoma, ATF2 downregulation upregulates MITF via SOX10, transforming melanoma into a metastatic phenotype.^220^ SOX10 expression is also modulated by factors like SOX9 phosphorylation by AKT,^221^ SLK deletion,^222^ MAPK signaling,^223^ or recurrent open chromatin domains.^224^ The SOX10‐MIR204‐ATF2 positive FBL can act as a toggle switch (Figure 6A), resulting in bistability in gene expression and regulating the switch between tumor phenotypes (i.e., two steady states in gene expression, each corresponding to a phenotype). Melanoma can exhibit three cell states: melanocytic, mesenchymal, and an intermediate state.^122^ Similar intermediate states are seen in EMT, previously considered binary, in pancreatic and epidermoid carcinoma.^225^, ^226^

Here, we explain how the FBL could regulate the phenotypic switch between these melanoma cell states (Figure 6B). When ATF2 activity is low, SOX10 is highly expressed (phase 1‐ON), leading to high miR‐204 and MITF levels that make melanoma cells show a melanocytic or intermediate phenotype. Increased ATF2 activity downregulates SOX10 (phase 2), which reduces miR‐204, amplifying ATF2 activity through the positive FBL. This causes SOX10 to switch to a lower steady state (phase 2‐OFF), downregulating MITF and resulting in a mesenchymal phenotype.^220^, ^227^ If SOX10 remains at the OFF steady state, it cannot revert to the ON steady state even if ATF2 activity returns to the low level (phase 3‐OFF). Consequently, low SOX10 expression leads to a low MITF expression, retaining the tumor in the mesenchymal phenotype. SOX10 only returns to the ON steady state when ATF2 activity drops below its initial level (phase 4‐ON), upregulating miR‐204 and normalizing MITF expression, potentially reverting cells to a melanocytic phenotype (Figure 6C). This hysteresis phenomenon^228^ delays the phenotypic switching of melanoma regulated by ATF2. However, it still remains unknown whether such a phenotypic transition of melanoma cells from mesenchymal states to intermediate or melanocytic cell states is possible.^229^ Therefore, it is possible that melanoma cells are confined to the mesenchymal phenotype due to irreversible switching (Figure 6D). A more complicated scenario is coupled FBLs and FFLs mediated by SOX10 and miRNAs that could cause tristability in SOX10 expression, leading to complex transitions among melanoma cell states, as seen in EMT.^230^, ^231^, ^232^ There is ongoing debate on melanoma cell states, with some proposing a spectrum of states^233^ and others suggesting distinct phenotypes stabilized by FBLs.^234^ Our results support the latter, with MITF bistability sustaining specific cell states, as observed in the MITF, BRN2, and miR‐211 FBL.^235^ This suggests that distinct melanoma cell states are underpinned by SOX10‐mediated network motifs.

Taken together, we showed a FBL containing the interactions between SOX10 and miRNAs and elucidated its role in regulating the dynamics of gene expression. This suggests that the distinct melanoma cell states are underpinned by SOX10‐mediated network motifs.

## 4 DISCUSSION AND CONCLUSION

The variable expression patterns of SOX10 and MITF within melanoma tumors significantly influence gene regulatory networks and cellular behaviors. Single‐cell studies reveal heterogeneity in SOX10 and MITF expression, leading to distinct regulatory states that impact downstream signaling.^186^ SOX10 promotes melanoma cell proliferation and survival but can also induce a differentiated, less aggressive phenotype.^32^, ^35^, ^223^ Interestingly, SOX10 depletion sensitizes melanoma cells to immune‐mediated killing by cytokines like TNF‐α and IFN‐γ, highlighting its dual role in tumor biology and immune interactions.^214^ Given these complexities, further research is needed to clarify the interplay between SOX10 and miRNAs.

miRNAs are critical regulators of SOX10 and MITF expression in melanoma, but their off‐target effects complicate molecular mechanisms.^236^, ^237^ Strategies like cooperative miRNA pairs can achieve desired repression with reduced off‐target risks.^80^ Beyond miRNAs, other ncRNAs, such as long ncRNAs and circular RNAs, are integral to SOX10‐mediated regulatory networks, influencing melanoma progression.^144^ Future research on ncRNA‐SOX10 crosstalk will deepen our understanding of melanoma pathogenesis.

We explored the intricate regulatory relationship between SOX10, MITF, and miRNAs in melanoma through miRNA‐mediated network motifs, such as FFLs and FBLs. Using network biology and dynamical systems approaches, we quantitatively analyzed how miRNA‐mediated interactions regulate tumorigenesis and phenotypic plasticity of melanoma. These findings underscore the therapeutic potential of targeting SOX10, MITF, and miRNAs in melanoma due to their pivotal role in cancer progression and hallmark modulation. In conclusion, a network biology perspective is crucial for understanding melanoma's complex regulatory mechanisms. SOX10, MITF, and miRNAs are potential therapeutic targets for melanoma due to their widespread expression in tumor samples and their instrumental role in modulating the hallmarks of cancer.

## AUTHOR CONTRIBUTIONS

**Xin Lai:** Conceptualization; methodology; software; data curation; investigation; validation; formal analysis; supervision; funding acquisition; visualization; project administration; resources; writing – original draft; writing – review and editing. **Chunyan Luan:** Data curation; software; visualization; writing – original draft; writing – review and editing. **Zhesi Zhang:** Visualization. **Anja Wessely:** Writing – review and editing. **Markus V. Heppt:** Writing – review and editing. **Carola Berking:** Resources; writing – review and editing. **Julio Vera:** Writing – review and editing; supervision; resources.

## CONFLICT OF INTEREST STATEMENT

The authors declare no conflicts of interest.

## Supporting information


**Data S1.** Supporting Information tables.

**Data S2.** Supporting Information figures.

**Data S3.** Supporting Information data.

## Data Availability

All data generated or analyzed during this study are deposited and available at https://doi.org/10.5281/zenodo.14111211.
