# Supplementary material for: SOX10, MITF, and microRNAs: Decoding their interplay in regulating melanoma plasticity
Source: Int J Cancer. 2025 Jun 3;157(7):1277–93. doi: 10.1002/ijc.35499 (PMC12334912; doi:10.1002/ijc.35499)
Supplement: Supplementary file 2 — Data S2. Supporting Information figures. [file IJC-157-1277-s002.pdf]

Supplementary Materials

SOX10, MITF, and microRNAs: decoding their interplay in regulating melanoma plasticity

Xin Lai, Chunyan Luan, Zhesi Zhang, Anja Wessely, Markus V. Heppt, Carola Berking, Julio Vera

Contents

Supplementary Tables 2

Supplementary Files 2

Supplementary Figures 3

The Supplementary Files and Tables are available in separate files online.

Supplementary Tables

Supplementary Tables S1 – S3 are uploaded as separate excel files.

Supplementary Files

Supplementary File S1: CellDesigner xml file for Figure 1

Supplementary File S2: Cytoscape file for Figure 2

Supplementary Files S1 and S2 are uploaded as separate files.

Supplementary Figures

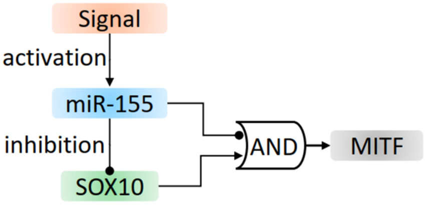

Phase	S	$\overline{miR}$	*SOX10*	*MITF*
1	1	0	0	0
2	0	1	0	0
3a	1	0	0	0
3b (off)	0	1	0	0 (delay)
3c	0	1	1	1
4 (on)	1	0	1	0

Phase	S	$\overline{miR}$	*SOX10*	*MITF*
1	0	1	1	1
2 (on)	1	0	0	0
3a	0	1	0	0
3b	1	0	0	0
4a (off)	0	1	0	0 (delay)
4b	0	1	1	0

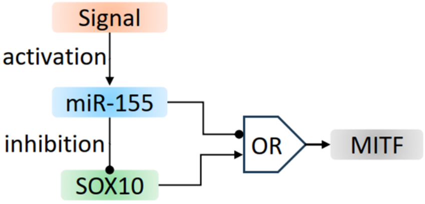

Phase	S	$\overline{miR}$	*SOX10*	*MITF*
1	1	0	0	0
2	0	1	0	1
3a	1	0	0	0
3b (off)	0	1	1	1
3c (on)	1	0	1	1 (delay)
4	1	0	0	0

Phase	S	$\overline{miR}$	*SOX10*	*MITF*
1	0	1	1	1
2a (on)	1	0	1	1 (delay)
2b	1	0	0	0
3a	1	0	0	0
3b	0	1	0	1
4 (off)	0	1	0	1

Supplementary Figure S1. The Boolean truth table of the AND and OR gate model for the MIR155-SOX10-MITF FFL. Each table corresponds to a dynamic plot shown in Figure 2. The phase column represents the four phases, with "on" and "off" corresponding to the states in the dynamic plot. The "delay" corresponds to the observed delay in MITF expression dynamics. $\overline{miR}$ represents the logic NOT expression of miR-155 (i.e. 0->1 or 1->0) because the miRNA inhibits the expression of MITF.

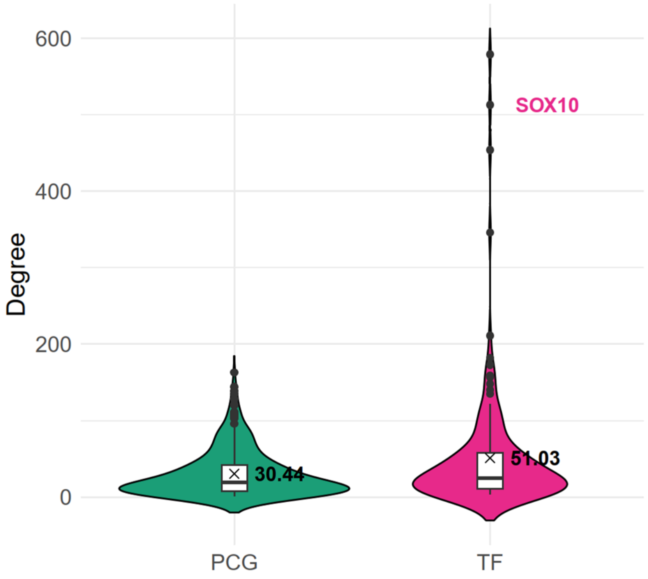

Supplementary Figure S2. The node degree distribution. The violin plot depicts the distribution of node degree among protein-coding genes (PCG) and transcription factors (TF) within the SOX10-centered network. The embedded boxplot illustrates the quartile distribution of the data, with thick horizontal lines representing the median value and crosses representing the mean value, for which the actual value is provided. The dots represent outliers, with SOX10 highlighted. The TFs have higher node degrees than the PCGs.
